# The ArcB kinase sensor participates in the phagocyte-mediated stress response in *Salmonella* Typhimurium

**DOI:** 10.3389/fmicb.2025.1541797

**Published:** 2025-02-11

**Authors:** Coral Pardo-Esté, Francisca Urbina, Nicolas Aviles, Nicolas Pacheco, Alan Briones, Carolina Cabezas, Vicente Rojas, Valentina Pavez, Yoelvis Sulbaran-Bracho, Alejandro A. Hidalgo, Juan Castro-Severyn, Claudia P. Saavedra

**Affiliations:** ^1^Laboratorio de Microbiología Molecular, Facultad de Ciencias de la Vida, Universidad Andrés Bello, Santiago, Chile; ^2^Laboratorio de Ecología Molecular y Microbiología Aplicada, Departamento de Ciencias Farmacéuticas, Facultad de Ciencias, Universidad Católica del Norte, Antofagasta, Chile; ^3^Laboratory of Entomology, Institute of Agri-Food, Animal and Environmental Sciences (ICA3), Universidad de O’Higgins, Rancagua, Chile; ^4^Centre of Systems Biology for Crop Protection (BioSaV), Institute of Agri-Food, Animal and Environmental Sciences (ICA3), Universidad de O’Higgins, San Fernando, Chile; ^5^Laboratorio de Patogénesis Bacteriana, Facultad de Medicina, Universidad Andres Bello, Santiago, Chile; ^6^Laboratorio de Microbiología Aplicada y Extremófilos, Departamento de Ingeniería Química, Universidad Católica del Norte, Antofagasta, Chile; ^7^Centro de Investigación Tecnológica del Agua y Sustentabilidad en el Desierto-CEITSAZA, Universidad Católica del Norte, Antofagasta, Chile

**Keywords:** ArcB, sensor kinase, *Salmonella*, reactive oxygen stress, HOCl, transcriptomic

## Abstract

The ArcAB two-component system includes a histidine kinase sensor (ArcB) and a regulator (ArcA) that respond to changes in cell oxygen availability. The ArcA transcription factor activates genes related to metabolism, membrane permeability, and virulence, and its presence is required for pathogenicity in *Salmonella* Typhimurium, which can be phosphorylated independently of its cognate sensor, ArcB. In this study, we aimed to characterize the transcriptional response to hypochlorous acid (HOCl) mediated by the presence of the ArcB sensor. HOCl is a powerful microbicide widely used for sanitization in industrial settings. We used wild-type *S.* Typhimurium and the mutant lacking the *arcB* gene exposed to NaOCl to describe the global transcriptional response. We also infected murine neutrophils to evaluate the expression levels of relevant genes related to the resistance and infection process while facing ROS-related stress. Our results indicate that the absence of the *arcB* gene significantly affects the ability of *S. Typhimurium* to grow under HOCl stress. Overall, 6.6% of *Salmonella* genes varied their expression in the mutant strains, while 8.6% changed in response to NaOCl. The transcriptional response associated with the presence of ArcB is associated with metabolism and virulence, suggesting a critical role in pathogenicity and fitness, especially under ROS-related stress. Our results show that ArcB influences the expression of genes associated with fatty acid degradation, protein secretion, cysteine and H_2_S biosynthesis, and translation, both *in vitro* and under conditions found within neutrophils. We found that protein carbonylation is significantly higher in the mutant strain than in the wild type, suggesting a critical function for ArcB in the response and repair processes. This study contributes to the understanding of the pathogenicity and adaptation mechanisms that *Salmonella* employs to establish a successful infection in its host.

## 1 Introduction

Two-component systems (TCSs) are critical for sensing bacterial stress response by regulating diverse pathogenicity and adaptation mechanisms. The key relevant functions of TCSs include environmental sensing, regulation of virulence, biofilm formation, antibiotic resistance, and quorum sensing (Capra and Laub, 2012; Mitrophanov and Groisman, 2008). Canonical TCSs consist of a histidine kinase (HK) and a response regulator (RR; Alvarez and Georgellis, 2022). Upon receiving a specific stimulus at the sensor domain, the HK phosphorylates and activates the RR (Brown et al., 2023). The anoxic redox control ArcAB TCS has been traditionally associated with the repression of aerobic respiration. It comprises the ArcB histidine kinase and the regulator ArcA (Iuchi and Lin, 1988; Iuchi et al., 1989; Malpica et al., 2006). The bacterial quinone pool is the main driver for ArcAB function, and its structure is highly conserved among the Enterobacteriaceae group (Georgellis et al., 2001; Federowicz et al., 2014).

The ArcA function is relevant during transition periods when redox states change and the usage of other electron acceptors such as nitrate is predominant (Federowicz et al., 2014). It also regulates the catabolism of fatty acids, amino acids, and carbon and aromatic compounds; biofilm formation; acid-resistant pathogenesis; and transport, responding to low oxygen conditions and decreased iron levels (Brown et al., 2023; Federowicz et al., 2014; Park et al., 2013; Shalel-Levanon et al., 2005; Liu and De Wulf, 2004; Pardo-Esté et al., 2019; Pardo-Esté et al., 2018). The other component of the TCS is the ArcB sensor kinase, which has an atypical configuration (Iuchi et al., 1989) and is able to sense the oxygen consumption rate (Nochino et al., 2020). Its phosphorylated form catalyzes transphosphorylation of the RR ArcA in response to changes in the redox state (Georgellis et al., 2001; Kwon et al., 2000; Rolfe et al., 2011). Theoretical models proposed that D-lactate and other metabolites produced during anaerobic metabolism may bind to the HK and could directly influence its activation (Padilla-Vaca et al., 2023).

The RR ArcA can be activated independently of ArcB under oxidizing conditions (Zhou et al., 2021). Our research team previously determined that the *in vitro* hypochlorous acid (HOCl) response mediated by ArcA was independent of its cognate sensor ArcB (Cabezas et al., 2021). The transcriptional factor ArcA partly mediates the ability of the bacterial pathogen *Salmonella* to resist reactive oxygen species (ROS)-induced stress, which phagocytes use as a microbicide after engulfing its target (Pardo-Esté et al., 2019; Pardo-Esté et al., 2018). Additionally, the ArcA function in the adaptation to ROS-related stress is present in other pathogens, suggesting that the response to microbicide-toxic compounds is a part of the ArcA regulon (Zhou et al., 2021; Loui et al., 2009; Lv et al., 2023).

The ArcB sensor is part of the regulatory networks, and its function is related to other systems such as RpoS-RssB; for example, it influences the phage shock protein (Psp) system in *Escherichia coli* cells (Jovanovic et al., 2006). ArcB is involved in the expression of the Type III secretion system in *Vibrio parahaemolyticus* (Zhang et al., 2023) and motility in *V. cholerae* and *S. marcescens* (Zhang et al., 2018; Wölflingseder et al., 2024). ArcB is also related to aerobic growth control in *Actinobacillus actinomycetemcomitans* under iron limitation functioning in conjunction with LuxS (Fong et al., 2003), although its participation during the bacterial ROS-related response remains to be elucidated.

In this study, we aimed to describe the transcriptional response that is dependent on the presence of the ArcB protein during the response of *Salmonella* to ROS-related stress. We describe the ArcB-mediated response of *Salmonella* against the neutrophil-induced stress, measured as cell damage and transcriptional response. This study contributes to the understanding of bacterial adaptation to a commonly used disinfectant and is relevant in the context of the current scenario of the emergence of multidrug-resistant *Salmonella* serotypes in clinical and industrial settings.

## 2 Methods

### 2.1 Ethics statement

Animals used in this study were maintained and manipulated following the recommendations in the Guide for the Care and Use of Laboratory Animals of the U.S. National Institutes of Health and the approved biosafety and bioethics protocol by the Universidad Andrés Bello Bioethics Committee, Protocol 06/2016 (FONDECYT Grant #1160315).

### 2.2 Bacterial strains and growth conditions

The *Salmonella* Typhimurium 14028 s parental strain and the Δ*arcB* mutant were maintained on LB agar plates in aerobiosis unless otherwise indicated. Cells were grown aerobically with shaking in LB medium at 37°C until reaching an OD_600_ of 0.4. The wild-type (WT) *S. enterica* serovar Typhimurium 14028 s was facilitated by Dr. Guido Mora (ATCC strain), and the Δ*arcB* strain was obtained previously (Morales et al., 2012). Furthermore, the Δ*arcB/*pBR::*arcB*, complemented with plasmid pBR322 containing the promoter and coding regions for *arcB*, was evaluated to measure viability and virulence as previously determined (Liu and De Wulf, 2004).

### 2.3 Minimal inhibitory concentration and growth rate

The bacterial strains were cultured overnight in LB medium at 37°C with aeration and shaking at 120 rpm. Minimal inhibitory concentration (MIC) assays for NaOCl were performed for both strains. Briefly, each microplate well containing dilution of NaOCl (from 0.1 to 25 mM) in LB medium was inoculated with the corresponding bacterial cultures in a 1:20 ratio. The plates were incubated at 37°C for 48 h with constant agitation, and OD_600_ values were measured using an Infinite 200 PRO microplate reader (TECAN, Inc.). For growth evaluation, two sets of flasks containing LB medium (one control and one supplemented with 1 mM NaOCl) were inoculated (1:100) with the grown strains (*S.* Typhimurium 14028s and Δ*arcB*), and the growth was monitored through CFU counts by taking 20 μl aliquots every 30 min and plated onto LB agar plates, which were subsequently incubated overnight at 37°C. This process was carried out for 16 h under the aforementioned growth conditions. Colony-forming units per milliliter (CFU/ml) were determined and transformed into Log10 CFU/ml. Data fitting and growth rate calculation were performed using the DMFit version of COMBASE’s Excel macro, applying the Baranyi and Roberts equation (Baranyi and Roberts, 1994).

### 2.4 Transcriptomic analysis

Overnight cultures of *S.* Typhimurium 14028s and Δ*arcB* strains were used to inoculate flasks with fresh LB medium (1:100) and grown at 37°C with 120 rpm agitation until an OD_600_ of ~0.4, at which point they were exposed to 1 mM NaOCl for 20 min, followed by a total RNA extraction from the harvested cells using the RNeasy Mini Kit (QIAGEN) following the manufacturer’s instructions. RNA integrity was assessed by 1.0% agarose gel electrophoresis, and quantification and quality were verified spectrophotometrically based on the OD_260/280_ ratio. The RNA was treated with 2 U of DNase I (Roche) for 1 h to remove contaminant DNA. To ensure no carry-over DNA in the samples, we routinely performed polymerase chain reaction (PCR) amplifications using primers for bacterial 16 s rRNA and found no product using the RNA extract as a template. Next, the total RNA was sent to Macrogen Inc. (Seoul, South Korea) for rRNA depletion (using the Ribo-Zero Plus Microbiome rRNA Depletion Kit; Illumina, Inc.), single-end (150 bp) cDNA library construction (using the TruSeq mRNA Library Prep Kit; Illumina, Inc.), and sequencing (on a HiSeq 2,500 platform; Illumina, Inc.). The RNA-seq raw data are available in the NCBI SRA database under accession numbers SRR9188681 and SRR9188682 (Bioproject PRJNA357075). Raw data quality control was accomplished using FastQC v0.11.8 (Andrews, 2010) followed by filtering and trimming with PRINSEQ v0.20.4 (Schmieder and Edwards, 2011) with the parameters 100 bp, 0 N, and <Q20 thresholds. The *S. enterica* subsp. enterica serovar Typhimurium strain 14028s reference genome (GenBank: GCA_000022165.1) was used as a reference to map the reads with Bowtie2 v2.3.5 (Langmead and Salzberg, 2012). The counts of reads that map against *Salmonella* ORFs were obtained using HTSeq v0.11.2 (Anders et al., 2015). The resulting matrix of counts was used to estimate differential gene expression using a normalization method implemented in the edgeR Bioconductor R Package (Robinson et al., 2010). The global expression patterns of the Δ*arcB* strain under control conditions and challenge with NaOCl (1 mM) were determined based on the 14028s (WT) strain expression patterns under the corresponding conditions. The results were filtered statistically (FDR ≤ 0.05) and biologically (LogFC ±2) to determine the list of genes with significant changes, whose functions were identified using UniProtKB (UniProt Consortium, 2023). Pathways and Gene Ontology (GO) enrichment analysis were carried out in R-base using the UniProtKB annotations for the differentially expressed genes. These were visualized using ggplot2 and pheatmap R packages (Kolde, 2019; Wickham, 2016).

### 2.5 Obtaining mouse bone-marrow-derived neutrophils

Female C57BL/6 mice (7–8 weeks old) were kept in plastic cages in a temperature-controlled environment (22–24°C) and were used to extract bone marrow as previously described (Swamydas and Lionakis, 2013). Then, bone marrow-derived neutrophils (BMDNs) were obtained using the mouse “Neutrophil Isolation Kit” (Miltenyi Biotec) following the manufacturer’s instructions. On average, 800,000 neutrophils/ml with approximately 85% viability were obtained for each replicate; these cells were positive for CD11b and Ly6G, as determined using flow cytometry. The viability of neutrophils was monitored throughout the experiments using trypan blue staining. Non-adherent BMDNs were maintained in RPMI medium 1,640 supplemented with 10% of FBS and 1X of Pen/Strep 100X antibiotics to avoid contamination.

### 2.6 Gentamicin protection assay

Cell infection assays were conducted using *S.* Typhimurium 14028s and its isogenic derivative Δ*arcB*. Bacteria were grown under microaerophilic conditions by adding an overlay of 500 μl of sterile mineral oil as a barrier to oxygen with no agitation until reaching an OD_600_ of 0.2. Prior to infection assays, bacteria were centrifuged (13,000 rpm, 5 min) and resuspended in 1 ml of cell RPMI culture medium supplemented with 10% FBS; as a result, the concentration of bacteria used to infect was 5×10^8^ bacteria/ml. The infected cells were stained with trypan blue to determine cell viability. Non-adherent murine neutrophils were kept in 15 ml falcon tubes and at a multiplicity of infection of 1:100. After 1 h incubation in 5% CO_2_ at 37°C, by triplicate the cells were centrifuged 5 min at 1,500 rpm and lysed with deoxycholate (0.5% w/v in PBS), serially diluted (10-fold) in PBS. Finally, the cells were used for RNA extraction and in parallel plated onto LB agar plates to obtain the CFU of each strain at 1 h post-infection (hpi). The remaining infected cells were washed three times (with 5 min centrifugation at 1,500 rpm intervals each wash) with sterile PBS and incubated in 5% CO_2_ at 37°C for 2 h with 100 μl cell medium plus 200 μg ml^−1^ gentamicin to kill extracellular bacteria. At 3 hpi, the medium was removed, and the cells were washed twice (with 5 min centrifugations at 1500 rpm intervals each wash) with PBS and lysed with sodium deoxycholate (0.5% w/v in PBS) and used for RNA extraction. In parallel, cell lysates were 10-fold serially diluted in PBS and plated onto LB agar plates to obtain the CFU counts at 3 hpi.

### 2.7 Total RNA extraction from NaOCl-treated Bacteria and infected phagocytes

RNA was obtained from bacteria recovered from infected BMDNs following the protocol previously described in Pardo-Esté et al. (2019), with slight modifications. Briefly, 10^7^ bacteria/ml grown in microaerophilic conditions were incubated with BMDN cells separately for 3 h. At 1 and 3 h post-infection (pi), cells were harvested, washed twice with PBS, and lysed with sodium deoxycholate (0.5% w/v in PBS). One sample was used as a bacterial viability control and was plated on LB plates. RNA extraction was performed using the E.Z.N.A Total RNA kit 1 de Omega Bio-Tek following the manufacturer’s instructions. RNA was suspended in 30 μl of nuclease-free water and its integrity was assessed by 1.0% agarose gel electrophoresis. Its concentration and quality were verified spectrophotometrically by the OD_260/280_ ratio. The RNA was treated with 2 U of DNase I (Roche) for 1 h to remove contaminant DNA. To ensure no carry-over DNA in the samples, we routinely performed PCR amplifications using primers for bacterial 16 s rRNA and found no product using the RNA extract as a template.

### 2.8 Transcriptional expression (qRT-PCR) from phagocyte-associated *Salmonella*

RNA extracted from phagocytized bacteria was used to obtain cDNA following the protocol previously described in Pardo-Esté et al. (2019). Briefly, the sample was treated at 37°C for 1 h in a 25-μl mixture containing 2.5 pmol of Random Primers (Invitrogen), 10 μl of template RNA (5 mg), 0.2 mM dNTPs, 1 μl of sterile water, 4 μl of 5 × buffer (250 mM Tris–HCl pH 8.3, 375 mM KCl, 15 mM MgCl_2_, and 10 mM DTT), and 200 U of reverse transcriptase (Invitrogen). The primers used for qRT-PCR are listed in Supplementary Table S1. Following the quantification of relative gene expression using the Brilliant II SYBR Green QPCR Master Reagent and the Mx3000P detection system (Stratagene), the qRT-PCR mixture (20 μl) containing 1 μl of the cDNA template and 120 nM of each primer under the following conditions: 10 min at 95°C, followed by 40 cycles of 30 s at 95°C, 45 s at 58°C, and 30 s at 72°C. The transcription level was quantified using Brilliant II SYBR Green qPCR Master Mix (Agilent Technologies) in a real PCR system AriaMx (Agilent Technology). Fold-change expressions of target genes normalized by the expression of the 16 s gene selected in these experimental conditions were calculated as previously described (Pfaffl, 2001).

### 2.9 Myeloperoxidase activity and HOCl and H_2_O_2_ quantification

The enzymatic activity of the myeloperoxidase (MPO) enzyme was quantified using a Neutrophil Myeloperoxidase Activity Assay Kit (Cayman Chemical), as previously described in (Pardo-Esté et al., 2019) using the color intensity of 3,3′,5,5′-tetramethyl-benzidine (650 nm) as an indicator of the MPO activity (μmoles/min/ml). Briefly, infected BMDNs were incubated at 1 and 3 hpi before measuring color intensity, and the results were normalized to the total protein concentration in the samples. Furthermore, the negative control included non-infected neutrophils and free bacteria, in addition to the negative controls with MPO inhibitor (4-aminobenzhydrazide) provided by the kit. Additionally, HOCl levels were determined based on the bleaching quantification of the green fluorescent protein (GFP) as an indirect measure of the increased HOCl. The two bacterial strains used for the infection assays were transformed with the plasmid pGlo containing the GFP and maintained episomally by adding 50 mM arabinose to the growth media. Fluorescence was determined using a TECAN Infinite 200 PRO microplate reader (395 nm excitation, 509 nm emission). Controls included cells with dimethyl sulfoxide (DMSO), free bacteria, PBS buffer, and non-infected eukaryotic cells activated with latex beads. The levels of hydrogen peroxide were measured using an Amplex® Red Hydrogen Peroxide/Peroxidase Assay Kit (Thermo Fisher), following the manufacturer’s instructions. The kit included positive and negative controls and the reactives to carry out a calibration curve.

### 2.10 Protein carbonylation, lipid peroxidation, and total glutathione quantification

The Protein Carbonyl Colorimetric (Cayman Chemical), TBARS (Cayman Chemical), and Glutathione (Cayman Chemical) assay kits were used for measuring protein carbonylation, lipid peroxidation, and total glutathione, respectively, following the manufacturer’s instructions, in BMDNs infected by *S.* Typhimurium 14028s and Δ*arcB* separately, as described above, and measurements for each indicator were performed at 1 and 3 hpi, respectively. In all cases, negative controls of non-infected phagocytes and free bacteria were used for normalization.

### 2.11 Statistical analyses

To determine statistical significance in gene expression and oxidative damage markers, we performed comparisons for each time point using one-way ANOVA with *α* = 0.05 with Tukey’s correction, comparing mutant strains with a wild-type strain separately at 1 and 3 hpi using R-base (R Core Team, 2020).

## 3 Results

**Figure 1:**
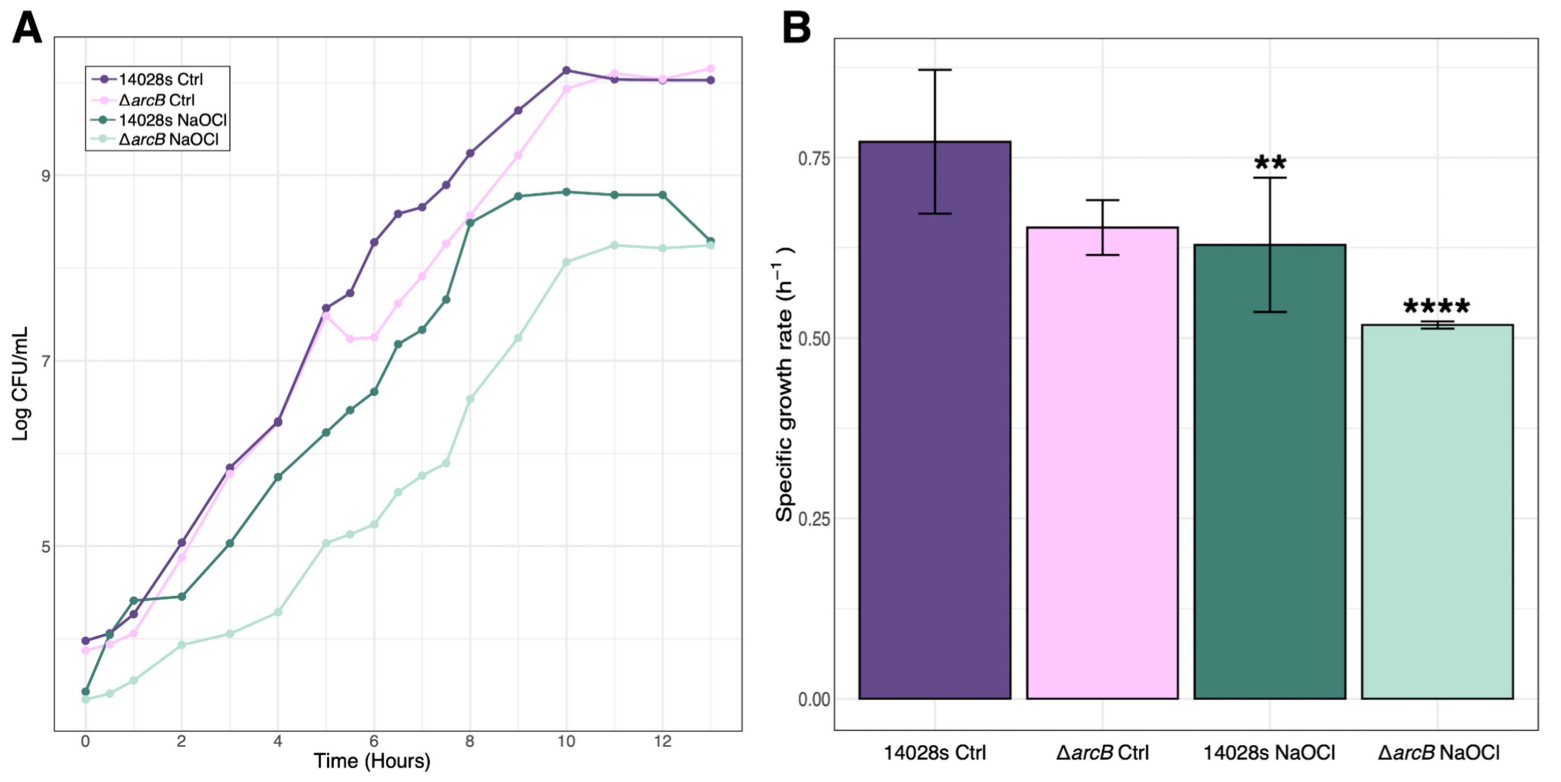
*Salmonella* Typhimurium growth in response to NaOCl. **(A)** Growth curves for the 14028s and Δ*arcB* mutant strains with and without 1 mM NaOCl treatment. **(B)** Specific growth rate for the parental 14028s and Δ*arcB* mutant strains under control conditions and facing NaOCl challenge. An analysis of variance (ANOVA) was performed with a significance level of *p* < 0.05, followed by Tukey’s multiple comparison test to determine differences between groups. The values represented are the mean ± standard error from three independent samples and three biological replicates.

The wild-type *S.* Typhimurium 14028s strain is able to resist up to 4 mM NaOCl (Pardo-Esté et al., 2019), while the Δ*arcB* mutant can only survive up to 2 mM of the toxic compound. Additionally, we found that there is a statistically significant decrease in bacterial growth when facing the toxic compound in the absence of the *arcB* mutant. This phenotype is recovered as previously found (Pardo-Esté et al., 2018). The untreated strains of *S.* Typhimurium 14028s, both the parental and the Δ*arcB* mutant, maintained constant specific growth rates (Figure 1). In contrast, treatment with NaOCl had a significant effect on the growth of both strains. This effect was more pronounced in the Δ*arcB* strain, which exhibited the lowest μmax when treated with NaOCl.

**Figure 2:**
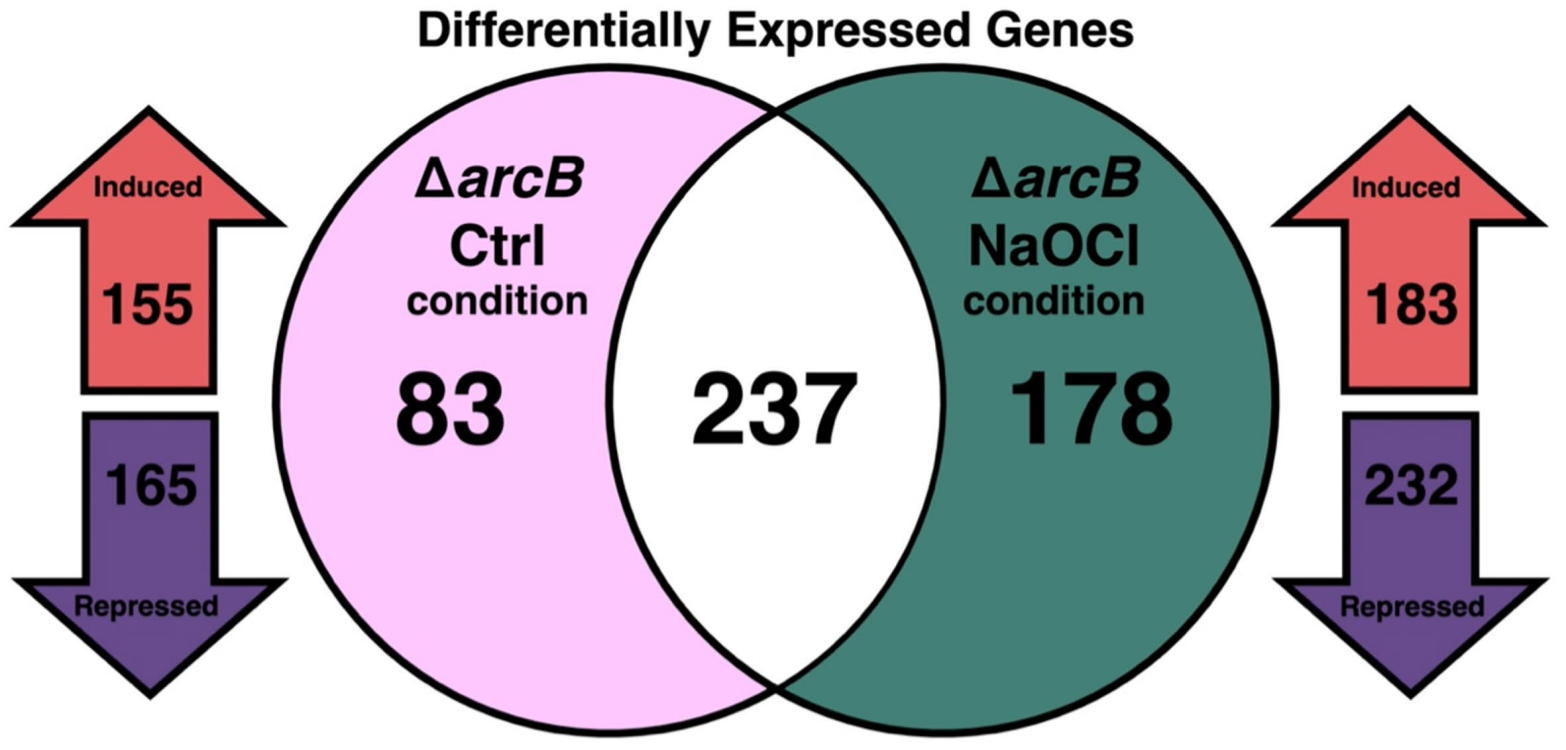
Transcriptomic patterns of the Δ*arcB* mutant strain. Statistical analysis of gene expression detected by RNA-seq. Venn diagram showing the total count of genes that change their expression in the Δ*arcB* strain regarding the 14028s parental strain under both conditions (Ctrl and NaOCl). The arrows show the counts of upregulated (induced in red) and downregulated (repressed in purple) genes under both conditions.

Transcriptome sequencing of the wild-type *Salmonella* Typhimurium 14028s and the Δ*arcB* mutant strain shows an average of 9.5 million reads per sample (depth 315X). Overall changes in transcription are detailed in Supplementary Figure 1. From a total of 4,851 genes in the *Salmonella* genome, 6.6% varied their expression on the Δ*arcB* strain compared to the wild-type parental strain under the control condition and 8.6% changed during the NaOCl challenge. In both conditions, there are more repressed genes than induced ones, suggesting a mainly suppressive influence of the ArcB protein activity (Figure 2).

**Figure 3:**
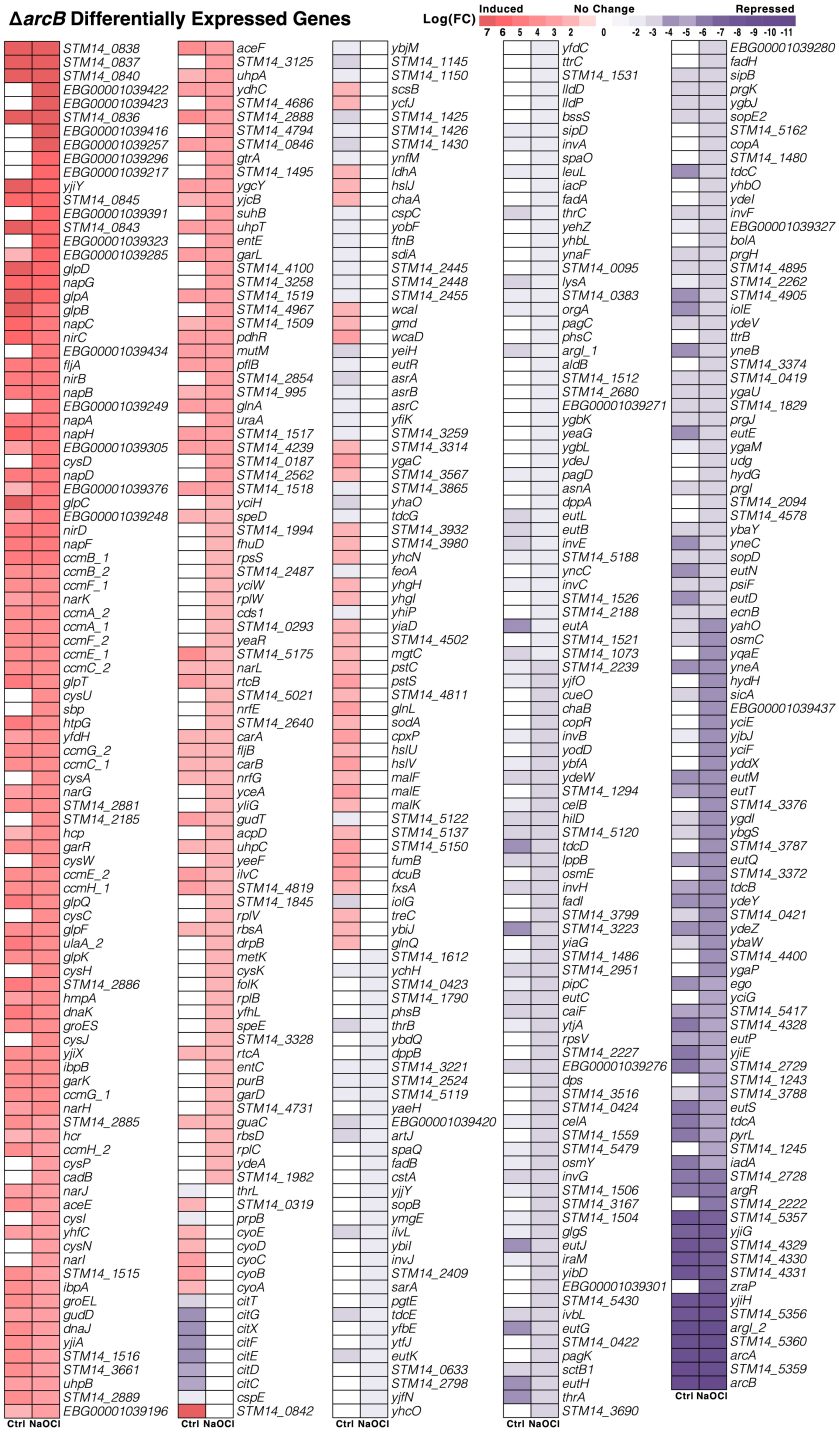
Differentially expressed genes in the Δ*arcB* mutant strain. Comparative transcriptomic patterns between the control and NaOCl conditions for the Δ*arcB* mutant strain and the 14028s parental strain. The expression level (LogFC) for each of the 498 identified genes (FDR ≤ 0.05) is shown correlatively to the heat scale at the top.

A total of 498 genes changed their expression in the Δ*arcB* mutant strain under both conditions, including a wide array of potential functions and also uncharacterized proteins (Figure 3). For instance, the sulfate/thiosulfate pathway for sulfur assimilation, nitrate reductase, and quinone oxidoreductase were upregulated. On the other hand, sensor histidine kinase, membrane shock, and osmoresponsive-associated genes were repressed when facing HOCl in the absence of ArcB. As expected, the *arcB* gene transcript was completely absent. However, critical processes like cysteine biosynthesis and translation were upregulated in the presence of the toxic compound, while protein secretion was downregulated in response to stress (Figure 3).

**Figure 4:**
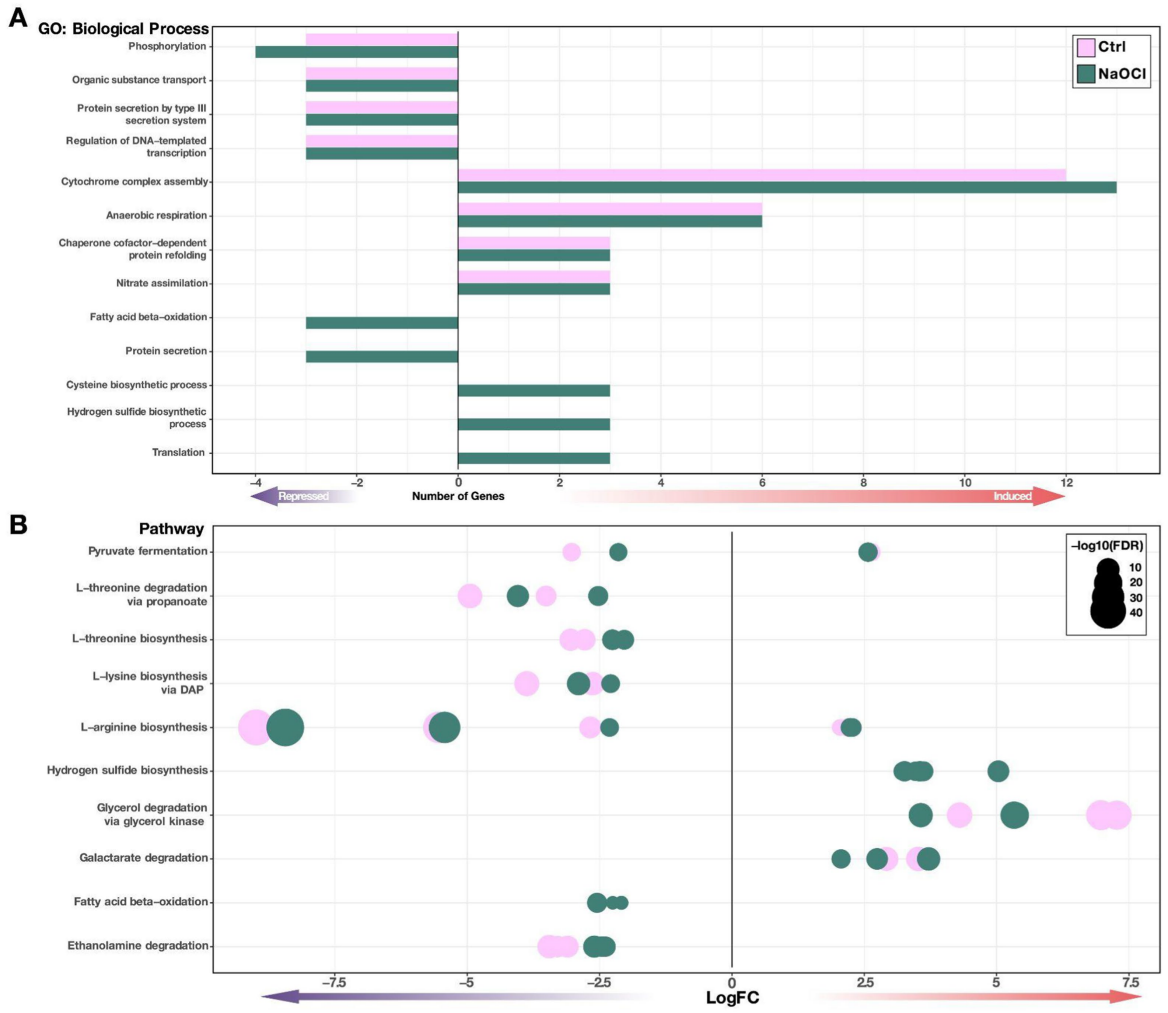
Functional enrichment analysis for the Δ*arcB* mutant strain. Functional classification of the differentially expressed genes in the Δ*arcB* mutant strain regarding the 14028s parental strain under Ctrl and NaOCl conditions. **(A)** Gene ontology (GO) enrichment is shown as the number of induced or repressed genes assigned to that category according to their identified function. These are exhibited as bars colored by conditions. **(B)** Enriched pathways are shown as dots representing individual genes, colored by conditions, sized by FDR value, and positioned on the X-axis according to the expression level (LogFC).

The role of ArcB in *Salmonella* functioning is diverse. Therefore, we aimed to group the genes with differential expression under both conditions into functional categories (biological processes) through an enrichment analysis (Figure 4) to get a better picture of the processes in which ArcB has influence. The GO analysis shows the biological processes associated with ArcB function under HOCl-induced stress, including fatty acid beta-oxidation and protein secretion, which are downregulated in the absence of the *arcB* gene. Fatty acid *β*-oxidation breaks down long-chain fatty acids into acetyl-CoA, producing reduced cofactors NADH and FADH₂ feeding into the electron transport chain, potentially leading to increased ROS production. ROS, in turn, can cause lipid peroxidation and damage key components of the β-oxidation pathway, disrupting fatty acid metabolism. Thus, there is a delicate equilibrium in which the ArcB function seems to be key. Regarding protein secretion systems, ROS can impair protein secretion by damaging secretory machinery and misfolding secreted proteins. Thus, cells may enhance the secretion of antioxidant enzymes and stress-responsive proteins to combat ROS damage, also highlighting the importance of ArcB.

On the other hand, processes such as cysteine and hydrogen sulfide biosynthesis and translation are induced during NaOCl treatment (Figure 4A). Cysteine plays a central role in ROS detoxification and cellular defense against oxidative stress. It is a critical component in antioxidants like glutathione and participates in redox regulation through thiol–disulfide exchange reactions and serves as a precursor for hydrogen sulfide (H₂S), which has additional protective roles, as it serves as a direct scavenger of ROS.

To obtain more insight into the functional differential enrichment of the gene, we also determined the influence of ArcB function over different pathways and found some similarities with the GO analysis (Figure 4B). Pyruvate fermentation, amino acid transformations, and fatty acid oxidation are downregulated, while hydrogen sulfide biosynthesis and glycerol and galactarate degradation are upregulated under HOCl stress. For instance, glycerol and galactarate degradation would increase ROS concentrations as a result of electron transport chain activity. These results suggest that ArcB would shift metabolic pathways away from oxidative phosphorylation during stress, minimizing ROS generation as well as avoiding the generation of new targets for the toxic compounds.

**Figure 5:**
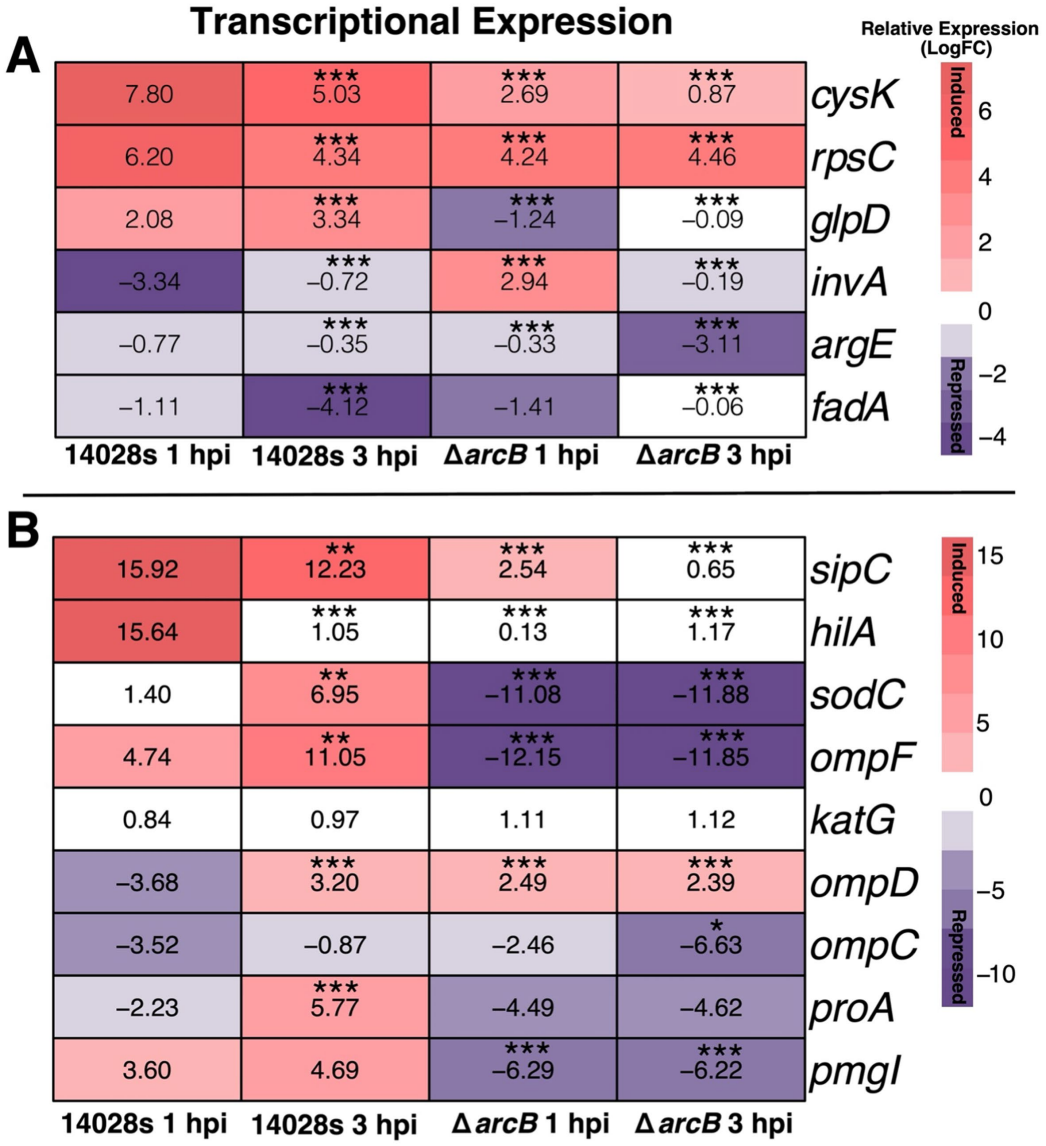
Transcriptional expression of selected genes. **(A)** associated with enriched pathways identified in the *in vitro* transcriptome and **(B)** involved in oxidative stress response and infection-related processes. Gene expression of the 14028s parental and Δ*arcB* mutant strains harvested from neutrophils at 1 and 3 hpi. Expression levels are exhibited as LogFC (relative to the 16S rRNA expression). Values represent the average of three independent experiments with three technical replicates each. Significance was assessed using one-way ANOVA with the Bonferroni correction (∗∗*p* < 0.01; ∗∗∗*p* < 0.001).

Neutrophils are part of the immune response to bacterial infection, and these cells use hypochlorous acid as their main toxic compound to induce bacterial death. In this context, we aimed to quantify the expression of genes that can be associated with the ability of *Salmonella* to survive inside phagosomes despite the presence of high concentrations of ROS-inducing compounds. Thus, we aimed to examine the expression of selected genes involved in pathways and biological processes that exhibited differential expression in the mutant bacteria lacking the *arcB* gene. We found that in bacteria harvested from infected neutrophils, the transcriptomic response (i.e., *argE, cysK, glpD, invA, rpsC*, and *fadA*) is also influenced by ArcB, as there is a statistically significant difference in the expression levels between the wild-type and the mutant strains (Figure 5A).

Additionally, our results show that the transcriptional expression of genes associated with membrane permeability, metabolism, virulence, and detoxification are influenced by the presence of *arcB*, which are key mediators for bacterial resistance increasing cell stability, prioritizing metabolic pathways, and activating protective mechanisms. In particular, those that are statistically significantly downregulated are the *sipC, sodC*, *ompF, ompC, proA*, and *pmg* genes, while the gene codifying for the major porin *ompD* is upregulated (Figure 5B).

The cell invasion protein *sipC* gene is induced during infection, as expected. In the mutant strain, the expression of this gene is seven times lower than that in the wild-type strain. A similar pattern was observed for the invasion activator *hilA*. The expression of the *katG* catalase is not dependent on ArcB, but *sodC* is strongly repressed in the cells must find an equilibrium between entrance or required solutes and expulsion of toxic compounds into the cells but also being able to secrete them, explaining the dynamics observed in the expression of *ompD, ompC* and *ompF,* associated with passive diffusion of nutrients and small molecules, that would also have a potential role as antigens during infection.

**Figure 6:**
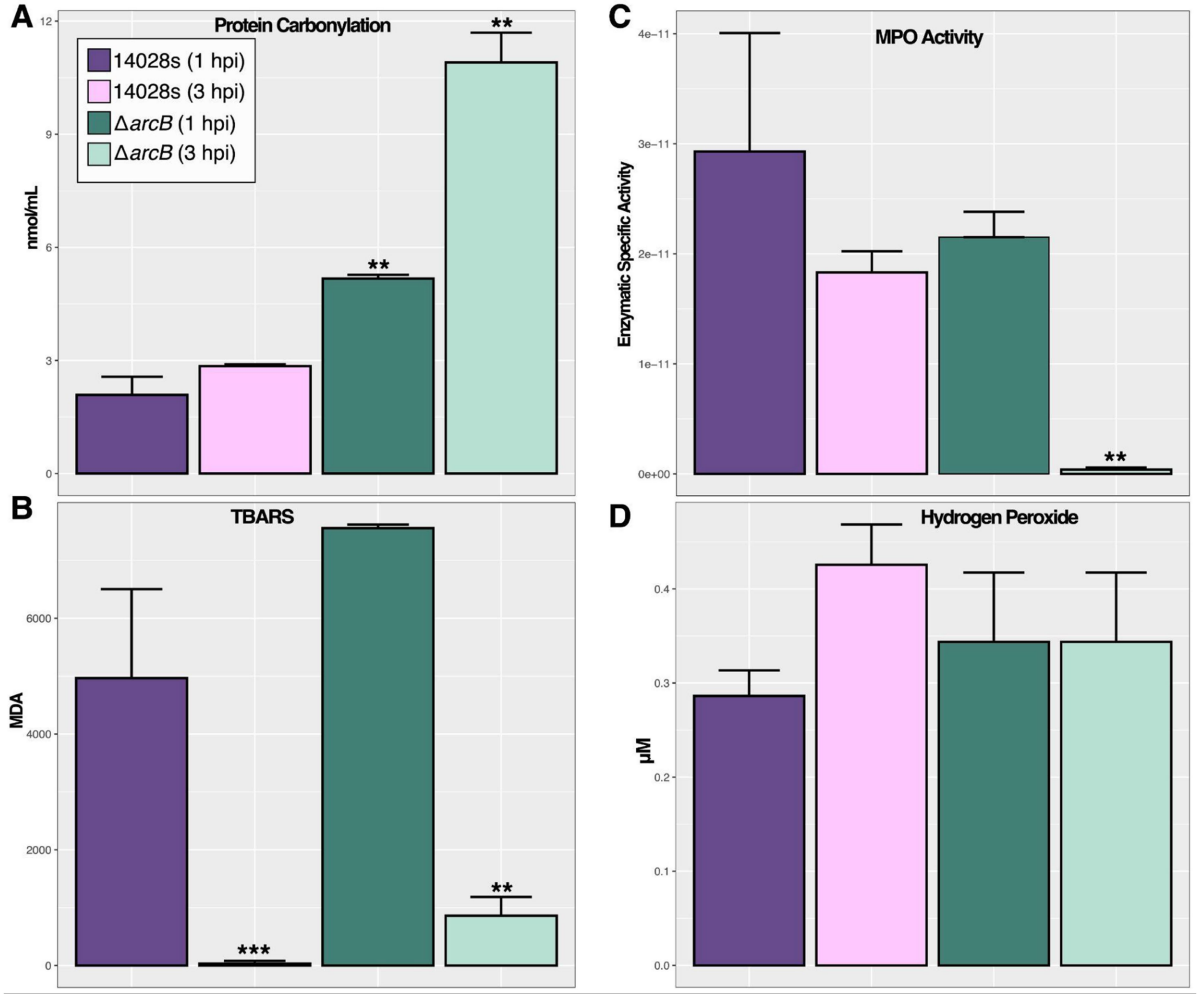
*Salmonella* oxidative stress indicators in BMDNs at 1 and 3 hpi. **(A)** Protein carbonylation as an indicator of oxidative damage. **(B)** Thiobarbituric acid reactive substances (TBARS) as an indicator of membrane oxidative damage. **(C)** Myeloperoxidase enzyme-specific activity measured as the protein units (μmol/ml) normalized by total protein concentration. **(D)** Total hydrogen peroxide accumulation as an indicator of cellular ROS status. These were measured on cells harvested from infected BMDNs at 1 and 3 hpi. The significance was calculated regarding the 14028s parental strain at 1 hpi, using one-way ANOVA with Tukey’s correction (∗*p* < 0.05; ∗∗*p* < 0.01; ∗∗∗*p* < 0.001). Values represent the average of five independent experiments with three technical replicates each.

To determine the physiological response of each strain under conditions faced during the neutrophil infection, we selected five approaches: protein carbonylation (Figure 6A) and lipid peroxidation (Figure 6B) to quantify the damage on the bacterial cells; myeloperoxidase enzyme activity (Figure 6C) to quantify the activation of key enzymes responsible for HOCl production in neutrophils; and H_2_O_2_ accumulation (Figure 6D) as another indicator of ROS accumulation. Furthermore, HOCl concentration was measured during the infection process to understand the temporal variation and associated effects (Supplementary Figure S2). We found that the bacteria were under constant influence of HOCl, with its concentration significantly increasing after 1 h of infection.

ROS causes cell damage that can be quantified as a measure of the ability of the bacteria to respond, repair, and survive under the effects of toxic compounds. Our results indicate that ArcB plays an important role in mediating this phenomenon as there are statistical differences between the wild-type control and the mutant strains in the context of cell damage and ROS concentrations. In particular, protein carbonylation was statistically significantly greater in the mutant strain (Figure 6A) than in the control, suggesting that the ArcB function may mediate the response or repair of damaged proteins. On the other hand, lipid peroxidation was generated after ROS accumulation in the wild-type and mutant strains, so the response or repair mechanisms may not be ArcB-induced (Figure 6B).

However, the activity of the myeloperoxidase enzyme is statistically less in the mutant strain under ROS stress at 3 hpi (Figure 6C), suggesting that bacteria lack the necessary immunological activation for the neutrophils that could affect the phagocytosis process. However, ROS and damage are accumulated in the bacterial cells suggesting a weaker response to the oxidative challenge (Supplementary Figure 2). Finally, hydrogen peroxide levels were maintained as expected during the assay in all evaluated strains (Figure 6D), as H_2_O_2_ is not the main toxic compound produced by neutrophils but many spontaneous reactions may occur in the phagosome.

Our results indicate that ArcB is directly involved in the response of *S.* Typhimurium to ROS-mediated stress *in vitro* and inside phagocytes. Here, we report that *in vitro* ArcB function is associated with fatty acid beta oxidation, protein secretion, cysteine biosynthesis, hydrogen sulfide biosynthesis, and translation in *Salmonella* under ROS-mediated stress. Moreover, the gene expression quantified in *Salmonella* infecting neutrophils also contributes to the understanding of how ArcB signals and participates in the regulatory network during infection and influences virulence, metabolism efficiency, and damage repair and survival. Thus, the sensor kinase ArcB is part of the regulatory network that actively participates in the activation of the response to ROS, in particular HOCl and the conditions found inside neutrophils during systemic infection.

## 4 Discussion

Our results suggest that in addition to its role as a global regulator for anaerobic growth of bacteria, the ArcAB system is also important for bacterial resistance to ROS in aerobic conditions, possibly through its influence on bacterial metabolism, especially amino acid and/or protein assimilation and synthesis (Loui et al., 2009). ArcB function is also associated with maintaining the redox state by influencing the production of antioxidant compounds. The ArcAB system promotes the survival of *S.* Typhimurium in macrophages and neutrophils and during systemic infection in mice (Pardo-Esté et al., 2018). Particularly, ArcA regulates the expression of several critical genes required to resist HOCl- and phagocyte-mediated stress (Pardo-Esté et al., 2019). The transcriptional response to HOCl mediated by ArcA is different from the one influenced by ArcB as demonstrated in this study. This non-cognate behavior was previously reported in vitro (Cabezas et al., 2021). Additionally, ROS damage would affect the quinone oxidation state and thus directly influence ArcB activation (Brown et al., 2023). Our results indicate that the absence of the ArcB function impairs bacterial survival under HOCl-induced stress, supporting the hypothesis that ArcAB is more closely associated with responding to the redox state of the cells rather than just oxygen availability (Federowicz et al., 2014; Toya et al., 2012).

Among the genes that induced their expression in response to NaOCl in the mutant strain are many associated with sulfate metabolism (*cysU*, *sbp*, *cysA*, *cysW*, *cysC*, *cysJ*, *cysP*) suggesting that ArcB could have a direct role, evidenced by the interplay of redox reactions (NADPH depletion), antioxidant systems (glutathione), and metabolic intermediates (i.e., sulfite). Additionally, virulence genes are dependent on the presence of *arcB*; for example, *suhB* is associated with O-antigen modifications that would be crucial during the phagocytic process *in vivo*, as well as siderophores (*entE*). Another critical function that is induced in response to ROS is nitrogen, amino acid (*nrfE, yceA*), and fatty acid metabolisms (*acpD*) that would be under strong regulation during the infection process where energy conservation and resources allocated within the cells would be determined in the bacterial survival.

On the other hand, genes that are downregulated in the mutant strain include *zraP*, which is strongly influenced by the function of ArcB and would participate in zinc homeostasis, ribosomal protection, and regulation of antioxidant enzymes such as superoxide dismutase and thus be critical for ROS defense. Furthermore, membrane permeability is crucial for regulating the flow of toxic compounds in and out of the cells; in this context, *ygaE* and *ygaP* are among the genes associated with this trait. Overall, the functions associated with ArcB are diverse and include virulence and intricate metabolic pathways that enable the cells to maintain redox status as well as basic cellular functions, highlighting the importance of this sensor kinase in the survival mechanisms used by *Salmonella* during ROS-induced stress.

Among the most critical activities found to be related to the ArcB function is fatty acid degradation. This pathway yields acetyl-coenzyme A (CoA), a critical precursor in bacterial metabolism (Nunn, 1986; Heath et al., 2002). Previous studies have determined that ArcA was involved in the regulation of the *fad* regulon regulating the machinery required for this process (Park et al., 2013; Cho et al., 2006). Additionally, it was postulated that regulation by the transcriptional factor FadR and ArcAB relies on the cAMP–CRP complex to activate transcription (Feng and Cronan, 2012). Here, we further determined that the absence of the *arcB* gene in *Salmonella* transcriptionally represses fatty acid degradation, suggesting that ArcB may promote this function.

Protein secretion is critical for bacterial virulence and pathogenicity (Green and Mecsas, 2016). There is ample evidence that ArcA regulates the function of the Type III secretion system and virulence protein secretion (Pardo-Esté et al., 2019; Wang et al., 2015). It has been previously demonstrated that ArcB is related to quorum sensing regulating T3SS in *V. parahaemolyticus* (Zhang et al., 2023). This study determined that the expression of genes related to this function is repressed in the mutant *arcB*, contributing to the hypothesis that ArcB may function in response to the redox state to promote bacterial virulence. It is expected that virulence response would be associated with ROS-related stress as the phagocytes aiming to eliminate bacteria use H_2_O_2_, O_2_, and HOCl as toxic compounds to attack the cells.

On the other hand, amino acid synthesis is critical for survival, especially during ROS-related stress when proteins might be damaged. While ROS can negatively impact amino acid synthesis by inhibiting enzymes and altering pathways, certain amino acids also play protective roles against oxidative stress by serving as precursors for antioxidants and modulating stress response pathways. Cysteine biosynthesis is a two-step process for incorporating the crucial sulfur atom into cellular components. Several molecular mechanisms regulate metabolisms, such as the LysR type, which positively regulate metabolic pathways, as well as Rrf2 and TetR, in addition to end-product inhibition (Guédon and Martin-Verstraete, 2006; Kredich, 2008). In the context of ROS-mediated stress, thioredoxin and glutathione are cysteine-derived proteins and are very important, as glutathione is also degraded to liberate cysteine (Guédon and Martin-Verstraete, 2006). The influence of ArcB in this case is repressing, as the function is promoted in the mutant strain.

Another related pathway that was significantly upregulated in the Δ*arcB* mutant is hydrogen sulfide (H_2_S) biosynthesis; this gas is produced by protein decomposition. The non-enzymatic pathway involves thiol-containing compounds such as glutathione (Shen et al., 2013; Yang et al., 2022). H_2_S is important for bacterial protection against antibiotics and the oxidative stress caused by them (Shatalin et al., 2011; Pal et al., 2018). It is also very relevant for regulating intestinal microbiota and virulence responses (Shen et al., 2013), thus contributing to the participation of ArcB in *S.* Typhimurium virulence. Finally, translation is an expected function to be influenced by ArcB, as it is part not only of the ArcAB regulation system but rather a network of collaborative signaling including Rpos-RssB, Fnr, and Crp (Wölflingseder et al., 2024; Perrenoud and Sauer, 2005). Thus, the absence of the *arcB* gene and its function would certainly affect translation efficiency.

The regulatory network that *Salmonella* implements as part of the response to the conditions found inside neutrophils includes ArcB, in particular functions critical for virulence, metabolism, and membrane permeability that are influenced by ArcB while facing the toxic compounds and other stressors found within the neutrophils. This highlights the importance of this sensor kinase as mediating the ability of *Salmonella* to survive and associates this molecule with other response regulators given its regulation pattern or activity is different from what was found in ArcA under the same conditions (Pardo-Esté et al., 2019) and in others (Brown et al., 2023).

Cellular damage in the Δ*arcB* mutant strain is more predominant on protein carbonylation level. As can be seen in the transcriptional response, the redox state and amino acid metabolism are closely related to the ArcB function, so it is expected that the mutant bacteria lack the ability to resist and repair damage caused by HOCl on proteins. Carbonylation causes irreversible and irreparable damage to proteins, which mostly affects the amino acids proline, arginine, lysine, and threonine, and has been used in organisms of all domains of life as an indicator of oxidative damage (Tamarit et al., 1998; Nyström, 2005). On the other hand, lipid damage seems to be harming both wild-type and mutant strains at similar levels, discarding a direct link with ArcB function.

These results contribute to the hypothesis that ArcB is a key mediator related to bacterial protein metabolism and repair. Furthermore, they shed light on the complex regulatory mechanisms enabling *Salmonella* to evade immunological attacks and withstand industrial antimicrobial treatments. This investigation further contributes to the understanding of the kinase sensor ArcB in the ability of *S.* Typhimurium to survive and bypass the innate immune system during its infection cycle by influencing the activity of fatty acid beta-oxidation and protein secretion, and cysteine and hydrogen sulfide metabolism and translation.

## 5 Conclusion

The ArcB sensor kinase is part of the complex regulatory network that enables *S.* Typhimurium to survive HOCl-related stress and phagocytosis to continue the infective cycle as well as industrial disinfection processes based on hypochlorous acid.

## Data Availability

The datasets presented in this study can be found in online repositories. The names of the repository/repositories and accession number(s) can be found in the article/Supplementary material.

## References

[ref1] Alvarez A. F. Georgellis D. (2022). The role of sensory kinase proteins in two-component signal transduction. Biochem. Soc. Trans. 50, 1859–1873. doi: 10.1042/BST20220848, PMID: 36398786

[ref2] Anders S. Pyl P. T. Huber W. (2015). HTSeq—a Python framework to work with high-throughput sequencing data. Bioinformatics 31, 166–169. doi: 10.1093/bioinformatics/btu638, PMID: 25260700 PMC4287950

[ref3] Andrews S. (2010). FastQC a quality-control tool for high-throughput sequence data. Available at: http://www.Bioinformaticsbabraham.ac.uk/projects/fastqc (Accessed July 7, 2024)

[ref4] Baranyi J. Roberts T. A. (1994). A dynamic approach to predicting bacterial growth in food. Int. J. Food Microbiol. 23, 277–294. doi: 10.1016/0168-1605(94)90157-0, PMID: 7873331

[ref5] Brown A. N. Anderson M. T. Smith S. N. Bachman M. A. Mobley H. L. T. (2023). Conserved metabolic regulator ArcA responds to oxygen availability, iron limitation, and cell envelope perturbations during bacteremia. MBio 14, e01448–e01423. doi: 10.1128/mbio.01448-23, PMID: 37681955 PMC10653796

[ref6] Cabezas C. E. Laulié A. M. Briones A. C. Pardo-Esté C. Lorca D. E. Cofré A. A.. (2021). Activation of regulator ArcA in the presence of hypochlorite in *Salmonella enterica* serovar Typhimurium. Biochimie 180, 178–185. doi: 10.1016/j.biochi.2020.11.009, PMID: 33188860

[ref7] Capra E. J. Laub M. T. (2012). Evolution of two-component signal transduction systems. Ann. Rev. Microbiol. 66, 325–347. doi: 10.1146/annurev-micro-092611-150039, PMID: 22746333 PMC4097194

[ref8] Cho B. K. Knight E. M. Palsson B. O. (2006). Transcriptional regulation of the fad regulon genes of *Escherichia coli* by ArcA. Microbiology 152, 2207–2219. doi: 10.1099/mic.0.28912-0, PMID: 16849788

[ref9] Federowicz S. Kim D. Ebrahim A. Lerman J. Nagarajan H. Cho B. K.. (2014). Determining the control circuitry of redox metabolism at the genome-scale. PLoS Genet. 10:e1004264. doi: 10.1371/journal.pgen.1004264, PMID: 24699140 PMC3974632

[ref10] Feng Y. Cronan J. E. (2012). Crosstalk of *Escherichia coli* FadR with global regulators in expression of fatty acid transport genes 7:e46275. doi: 10.1371/journal.pone.0046275 PMC3460868 23029459

[ref11] Fong K. P. Gao L. Demuth D. R. (2003). luxS and arcB control aerobic growth of *Actinobacillus actinomycetemcomitans* under iron limitation. Infect. Immun. 71, 298–308. doi: 10.1128/IAI.71.1.298-308.2003, PMID: 12496179 PMC143191

[ref12] Georgellis D. Kwon O. Lin E. C. (2001). Quinones as the redox signal for the arc two-component system of bacteria. Science (New York, N.Y.) 292, 2314–2316. doi: 10.1126/science.1059361, PMID: 11423658

[ref13] Green E. R. Mecsas J. (2016). Bacterial secretion systems: an overview. Microbiol. Spect. 4:2015. doi: 10.1128/microbiolspec.VMBF-0012-2015, PMID: 26999395 PMC4804464

[ref14] Guédon E. Martin-Verstraete I. (2006). “Cysteine metabolism and its regulation in Bacteria” in Amino acid biosynthesis ~ pathways, regulation and metabolic engineering. Microbiology monographs. ed. Wendisch V. F., vol. 5 (Berlin, Heidelberg: Springer).

[ref15] Heath R. White S. Rock C. (2002). Inhibitors of fatty acid synthesis as antimicrobial chemotherapeutics. Appl. Microbiol. Biotechnol. 58, 695–703. doi: 10.1007/s00253-001-0918-z, PMID: 12021787

[ref16] Iuchi S. Cameron D. C. Lin E. C. (1989). A second global regulator gene (arcB) mediating repression of enzymes in aerobic pathways of *Escherichia coli*. J. Bacteriol. 171, 868–873. doi: 10.1128/jb.171.2.868-873.1989, PMID: 2644240 PMC209676

[ref17] Iuchi S. Lin E. (1988). arcA (dye), a global regulatory gene in *Escherichia coli* mediating repression of enzymes in aerobic pathways. PNAS 85, 1888–1892. doi: 10.1073/pnas.85.6.1888, PMID: 2964639 PMC279886

[ref18] Jovanovic G. Lloyd L. J. Stumpf M. P. H. Mayhew A. J. Buck M. (2006). Induction and function of the phage shock protein extracytoplasmic stress response in *Escherichia coli*. J. Biol. Chem. 281, 21147–21161. doi: 10.1074/jbc.M602323200, PMID: 16709570

[ref19] Kolde R. (2019). Pheatmap: pretty Heatmaps. R package version 1.0.8 2015. Available at: https://CRAN.R-project.org/package=pheatmap (Accessed August, 2019).

[ref20] Kredich N. M. (2008). Biosynthesis of cysteine. EcoSal Plus 3:11. doi: 10.1128/ecosalplus.3.6.1.11 26443742

[ref21] Kwon O. Georgellis D. Lin E. C. (2000). Phosphorelay as the sole physiological route of signal transmission by the arc two-component system of *Escherichia coli*. J. Bacteriol. 182, 3858–3862. doi: 10.1128/JB.182.13.3858-3862.2000, PMID: 10851007 PMC94563

[ref22] Langmead B. Salzberg S. L. (2012). Fast gapped-read alignment with bowtie 2. Nat. Methods 9, 357–359. doi: 10.1038/nmeth.1923, PMID: 22388286 PMC3322381

[ref23] Liu X. De Wulf P. (2004). Probing the ArcA-P modulon of *Escherichia coli* by whole genome transcriptional analysis and sequence recognition profiling. J. Biol. Chem. 279, 12588–12597. doi: 10.1074/jbc.M313454200, PMID: 14711822

[ref24] Loui C. Chang A. C. Lu S. (2009). Role of the ArcAB two-component system in the resistance of *Escherichia coli* to reactive oxygen stress. BMC Microbiol. 9:183. doi: 10.1186/1471-2180-9-183, PMID: 19715602 PMC2748088

[ref25] Lv Q. Shang Y. Bi H. Yang J. Lin L. Shi C.. (2023). Identification of two-component system ArcAB and the universal stress protein E in Pasteurella multocida and their effects on bacterial fitness and pathogenesis. Microbes Infect.:105235. doi: 10.1016/j.micinf.2023.105235, PMID: 37802468

[ref26] Malpica R. Sandoval G. R. Rodríguez C. Franco B. Georgellis D. (2006). Signaling by the arc two-component system provides a link between the redox state of the quinone pool and gene expression. Antioxid. Redox Signal. 8, 781–795. doi: 10.1089/ars.2006.8.781, PMID: 16771670

[ref27] Mitrophanov A. Y. Groisman E. A. (2008). Signal integration in bacterial two-component regulatory systems. Genes Dev. 22, 2601–2611. doi: 10.1101/gad.1700308, PMID: 18832064 PMC2751022

[ref28] Morales E. H. Calderón I. L. Collao B. Gil F. Porwollik S. McClelland M.. (2012). Hypochlorous acid and hydrogen peroxide-induced negative regulation of *Salmonella enterica* serovar Typhimurium ompW by the response regulator ArcA. BMC Microbiol. 12:63. doi: 10.1186/1471-2180-12-63, PMID: 22545862 PMC3358236

[ref29] Nochino N. Toya Y. Shimizu H. (2020). Transcription factor ArcA is a flux sensor for the oxygen consumption rate in *Escherichia coli*. Biotechnol. J. 15:e1900353. doi: 10.1002/biot.201900353, PMID: 32383263

[ref30] Nunn W. D. (1986). A molecular view of fatty acid catabolism in *Escherichia coli*. Microbiol. Rev. 50, 179–192. doi: 10.1128/mr.50.2.179-192.1986, PMID: 3523188 PMC373063

[ref31] Nyström T. (2005). Role of oxidative Carbonylation in protein quality control and senescence. EMBO J. 24, 1311–1317. doi: 10.1038/sj.emboj.7600599, PMID: 15775985 PMC1142534

[ref32] Padilla-Vaca F. de la Mora J. García-Contreras R. Ramírez-Prado J. H. Vicente-Gómez M. Vargas-Gasca F.. (2023). Theoretical study of ArcB and its dimerization, interaction with anaerobic metabolites, and activation of ArcA. PeerJ 11:e16309. doi: 10.7717/peerj.16309, PMID: 37849831 PMC10578306

[ref33] Pal V. K. Bandyopadhyay P. Singh A. (2018). Hydrogen sulfide in physiology and pathogenesis of bacteria and viruses. IUBMB Life 70, 393–410. doi: 10.1002/iub.1740, PMID: 29601123 PMC6029659

[ref34] Pardo-Esté C. Castro-Severyn J. Krüger G. I. Cabezas C. E. Briones A. C. Aguirre C.. (2019). The transcription factor ArcA modulates Salmonella’s metabolism in response to neutrophil Hypochlorous acid-mediated stress. Front. Microbiol. 10:2754. doi: 10.3389/fmicb.2019.02754, PMID: 31866961 PMC6906141

[ref35] Pardo-Esté C. Hidalgo A. A. Aguirre C. Briones A. C. Cabezas C. E. Castro-Severyn J.. (2018). The ArcAB two-component regulatory system promotes resistance to reactive oxygen species and systemic infection by *Salmonella Typhimurium*. PLoS One 13:e0203497. doi: 10.1371/journal.pone.0203497, PMID: 30180204 PMC6122832

[ref36] Park D. M. Akhtar M. S. Ansari A. Z. Landick R. Kiley P. J. (2013). The bacterial response regulator ArcA uses a diverse binding site architecture to regulate carbon oxidation globally. PLoS Genet. 9:e1003839. doi: 10.1371/journal.pgen.1003839, PMID: 24146625 PMC3798270

[ref37] Perrenoud A. Sauer U. (2005). Impact of global transcriptional regulation by ArcA, ArcB, Cra, Crp, Cya, Fnr, and Mlc on glucose catabolism in *Escherichia coli*. J. Bacteriol. 187, 3171–3179. doi: 10.1128/JB.187.9.3171-3179.2005, PMID: 15838044 PMC1082841

[ref38] Pfaffl M. W. (2001). A new mathematical model for relative quantification in real-time RT-PCR. Nucleic Acids Res. 29:e45, 45e–445e. doi: 10.1093/nar/29.9.e45, PMID: 11328886 PMC55695

[ref39] R Core Team (2020). R: A language and environment for statistical computing. R foundation for statistical computing, Vienna, Austria. Available at: https://www.R-project.org/.

[ref40] Robinson M. D. McCarthy D. J. Smyth G. K. (2010). edgeR: a Bioconductor package for differential expression analysis of digital gene expression data. Bioinformatics 26, 139–140. doi: 10.1093/bioinformatics/btp616, PMID: 19910308 PMC2796818

[ref41] Rolfe M. D. Ter Beek A. Graham A. I. Trotter E. W. Asif H. M. Sanguinetti G.. (2011). Transcript profiling and inference of *Escherichia coli* K-12 ArcA activity across the range of physiologically relevant oxygen concentrations. J. Biol. Chem. 286, 10147–10154. doi: 10.1074/jbc.M110.211144, PMID: 21252224 PMC3060466

[ref42] Schmieder R. Edwards R. (2011). Quality control and preprocessing of metagenomic datasets. Bioinformatics 27, 863–864. doi: 10.1093/bioinformatics/btr026, PMID: 21278185 PMC3051327

[ref43] Shalel-Levanon S. San K. Y. Bennett G. N. (2005). Effect of oxygen, and ArcA and FNR regulators on the expression of genes related to the electron transfer chain and the TCA cycle in *Escherichia coli*. Metab. Eng. 7, 364–374. doi: 10.1016/j.ymben.2005.07.001, PMID: 16140031

[ref44] Shatalin K. Shatalina E. Mironov A. Nudler E. (2011). H2S: A universal defense against antibiotics in bacteria. Science (New York, N.Y.) 334, 6058, 986–6990. doi: 10.1126/science.1209855, PMID: 22096201

[ref45] Shen X. Carlström M. Borniquel S. Jädert C. Kevil C. G. Lundberg J. O. (2013). Microbial regulation of host hydrogen sulfide bioavailability and metabolism. Free Radic. Biol. Med. 60, 195–200. doi: 10.1016/j.freeradbiomed.2013.02.024, PMID: 23466556 PMC4077044

[ref46] Swamydas M. Lionakis M. S. (2013). Isolation, purification and labeling of mouse bone marrow neutrophils for functional studies and adoptive transfer experiments. JoVE 77:e50586. doi: 10.3791/50586, PMID: 23892876 PMC3732092

[ref47] Tamarit J. Cabiscol E. Ros J. (1998). Identification of the major oxidatively damaged proteins in *Escherichia coli* cells exposed to oxidative stress. J. Biol. Chem. 273, 3027–3032. doi: 10.1074/jbc.273.5.3027, PMID: 9446617

[ref48] Toya Y. Nakahigashi K. Tomita M. Shimizu K. (2012). Metabolic regulation analysis of wild-type and arcA mutant *Escherichia coli* under nitrate conditions using different levels of omics data. Mol. BioSyst. 8, 2593–2604. doi: 10.1039/c2mb25069a, PMID: 22790675

[ref49] UniProt Consortium (2023). UniProt: the universal protein knowledgebase in 2023. Nucleic Acids Res. 51, D523–D531. doi: 10.1093/nar/gkac1052, PMID: 36408920 PMC9825514

[ref50] Wang Q. Cen Z. Zhao J. (2015). The survival mechanisms of thermophiles at high temperatures: an angle of omics. Physiology 30, 97–106. doi: 10.1152/physiol.00066.2013, PMID: 25729055

[ref51] Wickham H. (2016). ggplot2: elegant graphics for data analysis. New York, NY: Springer-Verlag.

[ref52] Wölflingseder M. Fengler V. H. Standhartinger V. Wagner G. E. Reidl J. (2024). The regulatory network comprising ArcAB-RpoS-RssB influences motility in *Vibrio cholerae*. Mol. Microbiol. 121, 850–864. doi: 10.1111/mmi.15235, PMID: 38323722

[ref53] Yang Z. Wang X. Feng J. Zhu S. (2022). Biological functions of hydrogen sulfide in plants. Int. J. Mol. Sci. 23:15107. doi: 10.3390/ijms232315107, PMID: 36499443 PMC9736554

[ref54] Zhang C. Liu M. Wu Y. Li X. Zhang C. Call D. R.. (2023). ArcB orchestrates the quorum-sensing system to regulate type III secretion system 1 in *Vibrio parahaemolyticus*. Gut Microbes 15:2281016. doi: 10.1080/19490976.2023.2281016, PMID: 37982663 PMC10841015

[ref55] Zhang X. Wu D. Guo T. Ran T. Wang W. Xu D. (2018). Differential roles for ArcA and ArcB homologues in swarming motility in *Serratia marcescens* FS14. Antonie Van Leeuwenhoek 111, 609–617. doi: 10.1007/s10482-017-0981-9, PMID: 29139003

[ref56] Zhou Y. Pu Q. Chen J. Hao G. Gao R. Ali A.. (2021). Thiol-based functional mimicry of phosphorylation of the two-component system response regulator ArcA promotes pathogenesis in enteric pathogens. Cell Rep. 37:110147. doi: 10.1016/j.celrep.2021.110147, PMID: 34936880 PMC8728512

