# Supplementary figures and images for: The ArcB kinase sensor participates in the phagocyte-mediated stress response in Salmonella Typhimurium
Source: Front Microbiol. 2025 Feb 11;16:1541797. doi: 10.3389/fmicb.2025.1541797 (PMC11850377; doi:10.3389/fmicb.2025.1541797)

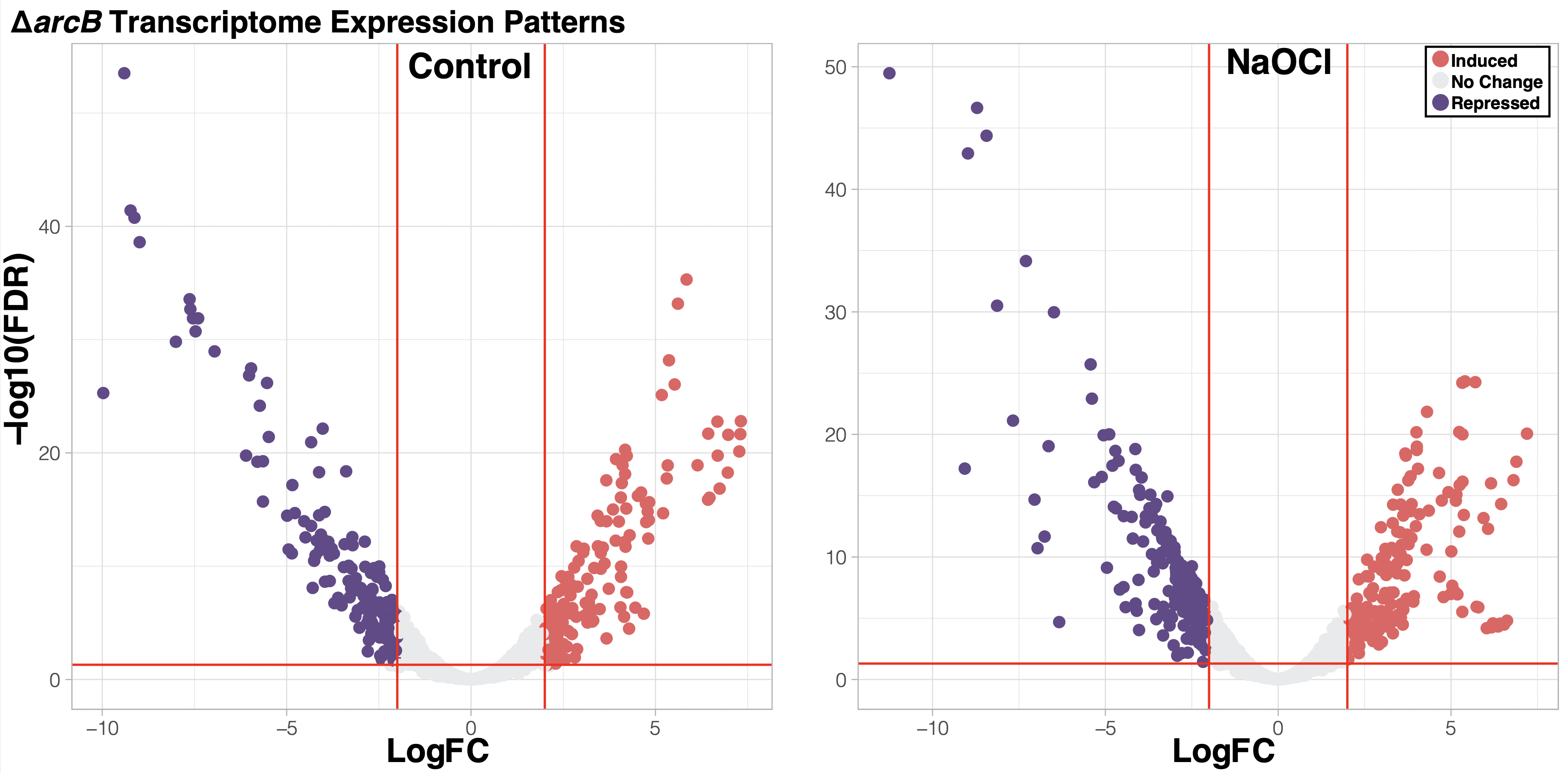

Supplement: Supplementary file 1 [file Image_1.PNG]

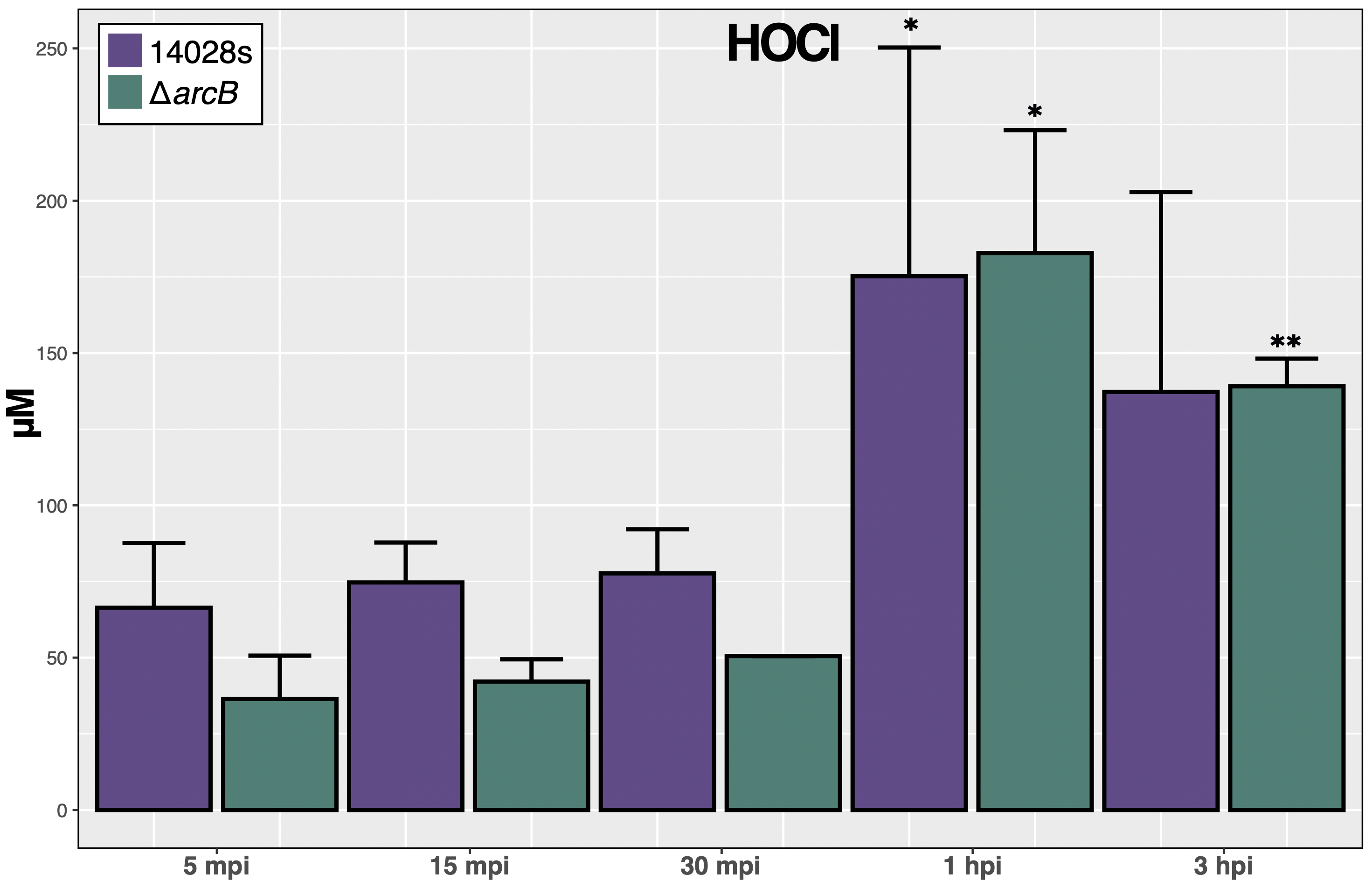

Supplement: Supplementary file 2 [file Image_2.PNG]
